# Affections of the Urinary Organs

**Published:** 1828-04

**Authors:** 


### AFFECTIONS OF THE URINARY ORGANS.

### Case I.—*Ruptured Urethra.*

William Taylor, ætatis forty-two, was admitted into St. Bartholomew's Hospital, October 20th, under the care of Mr. Earle, having fallen across the edge of a door on his perineum. Considerable bleeding took place from the urethra, and effusion into the scrotum and perineum opposite the bulb. He had not passed any urine for many hours. An attempt was made by the dresser to pass a catheter, without success; and Mr. Stanley, who was passing through the ward, was requested to see him. After some time he succeeded in passing a small elastic gum catheter into the bladder, and some water mixed with blood was drawn off. Mr. S. distinctly felt the rupture in the urethra in passing the instrument, which was directed to be left in the bladder.

Mr. Earle saw the patient the following day, and found a tumor of the size of a large walnut rather to the left of the bulb. The scrotum was black with effused blood, but not much distended. Urine mixed with blood continued to flow through the catheter. He was largely bled from the arm, and twenty leeches were applied to the perineum.

He continued to go on favorably for some days, but on the 25th the catheter slipped out, and the patient attempted to reintroduce it, which caused some return of arterial bleeding. He had suffered during the preceding night with severe rigors and fever. Mr. Earle introduced a large catheter without difficulty, in doing which the laceration in the urethra was distinctly felt about the bulbous part. The shivering and fever returned at night, and the patient removed the catheter, which was followed by considerable hemorrhage.

On the following day he was very ill, with frequent disposition to shiver, and the tumor in the perineum had increased in size. A free incision was made through the tumor, and extended downwards towards the anus. The membranous part of the urethra was distinctly felt, but not opened, as the lacerated opening communicated directly with the upper part of the incision, and afforded a ready exit for the urine. The wound bled freely, and gave the patient much relief. From this time he was able to pass water without the assistance of the catheter, partly through the wound, but principally through the natural passage. The wound suppurated kindly, and speedily healed.

It was necessary for some time to pass bougies, to counteract the effect of the contraction at the cicatrised portion of the urethra.

---

Case II.—*Ruptured Urethra.*

William Barnet, ætatis thirty-six, was admitted September 13th, 1827, into St. Bartholomew's Hospital. He stated that, on the previous evening, he had fallen about fifteen feet, and struck the perineum across an iron bar. Violent hemorrhage took place from the urethra, and the scrotum and integuments became distended with blood, accompanied with severe pain. He sent for a surgeon, who made many attempts to pass a catheter without success. He was bled, and leeches were applied to the part; but he passed a night of great misery, and the following day was admitted into the hospital. His bladder was at this time to be felt above the pubes; no urine had passed since the accident, (sixteen hours,) nor for some hours before. His countenance was anxious, and expressive of much suffering; pulse 100, and full; perineum and scrotum much distended, and of a dark livid colour: there was no external wound.

Mr. Earle cautiously introduced a full-sized silver catheter, which passed readily down into a cavity filled with coagulum between the rectum and membranous part of the urethra. The finger, introduced into the rectum, readily detected the point of the catheter in this situation. Mr. E. immediately made a free incision opposite to the bulb of the urethra, and extended it parallel to the rupture to the extent of two inches. A quantity of coagulum and fresh blood escaped, and the catheter became apparent, passing through the ruptured opening at the upper part of the bulb. Mr. Earle attempted to introduce an elastic gum catheter from this part into the bladder, but not readily succeeding, he desisted from any effort. The finger, introduced into the wound, passed into a large cavity filled with coagulum. It did not appear that any urine had been effused. He was placed in a warm hip-bath, which encouraged the bleeding from the wound, and loosened some of the coagulum. Whilst in the bath, some urine flowed through the wound. He now became very faint, and was removed to bed; and the bleeding was restrained by the application of lint and cold cloths. Urine mixed with blood continued to dribble away. He passed a tranquil night, without any return of bleeding.

On the 15th, he passed about half a pint of urine voluntarily through the wound, which relieved him much.

On the 17th, he experienced difficulty in passing his water through the wound, and the dresser endeavoured to remove a coagulum which presented itself. This was followed by a return of arterial bleeding, which continued to flow through the greater part of the night, until the patient was alarmingly faint, requiring the administration of brandy and ammonia, with opium.

On the 18th, no urine had passed, but the bladder was not distended. He continued very faint, with a feeble intermitting pulse. On raising him upon a night-chair, he was able to pass water through the wound. Suppuration now began to take place, and no farther alarming symptoms occurred.

October 2d.—A good-sized metallic bougie, No. 12, was introduced. On reaching the situation of the wound, it met with some resistance, which was readily overcome, and the instrument passed on without difficulty into the bladder. On withdrawing the bougie, the patient passed some water through the natural passage.

The bougie was introduced every second day. The wound in the perineum now rapidly healed, and was closed by the 8th of October.

Mr. Earle remarks, the preceding cases afford a good illustration of the best method of treating these serious accidents, which are sometimes followed by the most disastrous consequences from the effusion of urine, without the most prompt and decided treatment on the part of the surgeon. Where there is reason to apprehend that the urethra has given way, either by ulceration or from the application of force, the most judicious plan is to make a free opening in the perineum, by which we at once secure a ready exit for the urine, blood, and matter, and prevent any extension of the mischief. This is far better than to persevere in endeavouring to introduce instruments into the bladder, by which the injury is often much increased, while the attempts prove abortive; and, even should they succeed, it frequently happens that the presence of the foreign body in the urethra keeps up the irritation and increases the malady. In many of these cases, where there is no external wound, and where the patient is ignorant of the nature of the injury, and the danger to be apprehended, it is often difficult to persuade him or his friends of the necessity for such an operation. Much decision and firmness are required on the part of the surgeon, who should act at once, or he may be too late to prevent extensive or even fatal effusion of urine. No possible danger is to be apprehended from the performance of the operation, which places the patient in a state of security, and enables nature to set about her process of reparation. The wounds always heal readily, if properly treated, and the external incision be of sufficient extent.

---

### Case III.—*Retention of Urine.*

H. Hoskin, æt. thirty-two, a sailor, admitted December 31st, 1827, into St. Thomas's Hospital, under Mr. Green, in robust health, caught, six weeks ago, a virulent gonorrhœa, for which he has taken a small quantity of medicine, which merely proved aperient. He continued his intemperance, and, about a fortnight before admission, the gonorrhœal discharge somewhat diminishing, the stream of urine was observed also to decrease, gradually becoming less, till yesterday morning, when the gonorrhœal discharge entirely ceased, and with it the power of voiding the urine, even in drops.

When admitted, (four o'clock p.m.) had passed no urine for forty hours; the attempts to do so very frequent and urgent; considerable swelling at lower part of abdomen; great tenderness and tension, with constant sense of "burning" in region of bladder; great thirst; hot and dry skin; anxious countenance; quick and hard pulse; constipation of bowels.

Immediately placed in a warm bath for one hour and a quarter, and forty-eight ounces of blood abstracted, which produced fainting, when a gentle attempt was made to pass the catheter; but, spasm being induced, and hæmaturia returning, it was not persevered in. On leaving the bath, about a quarter of a pint of urine was passed.

Ordered ℞. Ol. Ricini ℥ss.—Tinct. Opii Sedativ. m. xx. statim sum.

January 1st.—Somewhat better. Continued passing small quantities of urine till three o'clock this morning, when he fell asleep, and none has since passed. Less tenderness and tension of abdomen; bowels freely open; pulse more compressible.

Vespere.—Has passed no urine since morning: inflammatory symptoms increasing.

Warm bath and draught repeated.

The symptoms becoming more and more urgent, at one o'clock a.m. (January 2d,) Mr. Green was sent for, and at two proceeded to relieve him by operation.

Having placed the patient as for lateral operation of stone, Mr. G. introduced the male catheter down to a slight permanent stricture, which existed just where the urethra contracts at the bulb.

He then made an incision about an inch and a half along the raphé, not quite so high, however, as to reach the catheter, as that could not be effected without dividing part of the scrotum, which was unnecessary. Having found the urethra, he attempted to pass the catheter forward into the bladder, but, finding this impracticable, a female catheter was introduced through the opening, and a considerable quantity of urine drawn off. The male catheter was then carried by the side of the female catheter into the bladder, where it was permitted to remain. A rather large vessel was divided during the operation, and it was necessary (it being too deep-seated to secure by ligature,) to restrain the hemorrhage by pressure, which was continued one hour and a half.

In some remarks on the above case, Mr. Green observed that it presented a fair specimen of his treatment of spasmodic, combined with some degree of permanent stricture. 1st. The production of fainting and very gentle attempts to introduce the catheter. 2dly. If unsuccessful and inflammatory symptoms are present, (which he considers a good criterion as to the necessity for it,) at once proceed to the operation. 3dly. The operation itself. There are two conditions of parts for which he prefers operating in the perineum,—viz. when there is a severe permanent stricture, or extravasation. In this case, it will be observed, there was neither: the stricture was slight, and might have been relieved by bougies; but it being very probable a false passage had been formed before his admission, (and if so, bougies could not have been employed with security,) Mr. G. considered it as sufficient to direct his choice. He prefers making his incision along the raphé, from the comparative facility with which the urethra may be found, which at best, without the guidance of a staff, is accomplished with some difficulty.

---

